# Using mobile health technology to assess childhood autism in low-resource community settings in India: An innovation to address the detection gap

**DOI:** 10.1177/13623613231182801

**Published:** 2023-07-17

**Authors:** Indu Dubey, Rahul Bishain, Jayashree Dasgupta, Supriya Bhavnani, Matthew K Belmonte, Teodora Gliga, Debarati Mukherjee, Georgia Lockwood Estrin, Mark H Johnson, Sharat Chandran, Vikram Patel, Sheffali Gulati, Gauri Divan, Bhismadev Chakrabarti

**Affiliations:** 1 University of Reading, UK; 2 University of Nottingham, UK; 3 Indian Institute of Technology Bombay, India; 4 Sangath, India; 5 Samvedna Care, India; 6 Public Health Foundation of India, India; 7 The Com DEALL Trust, India; 8 Nottingham Trent University, UK; 9 University of East Anglia, UK; 10 University of London, UK; 11 Indian Institute of Public Health, Bengaluru, India; 12 University of East London, UK; 13 University of Cambridge, UK; 14 Harvard University, USA; 15 All India Institute of Medical Sciences, India; 16 India Autism Center, India; 17 Ashoka University, India

**Keywords:** Autism, digital health, global, LMIC

## Abstract

A diagnosis of autism typically depends on clinical assessments by highly trained professionals. This high resource demand poses a challenge in low-resource settings. Digital assessment of neurodevelopmental symptoms by non-specialists provides a potential avenue to address this challenge. This cross-sectional case-control field study establishes proof of principle for such a digital assessment. We developed and tested an app, START, that can be administered by non-specialists to assess autism phenotypic domains (social, sensory, motor) through child performance and parent reports. *N* = 131 children (2–7 years old; 48 autistic, 43 intellectually disabled and 40 non-autistic typically developing) from low-resource settings in Delhi-NCR, India were assessed using START in home settings by non-specialist health workers. The two groups of children with neurodevelopmental disorders manifested lower social preference, greater sensory interest and lower fine-motor accuracy compared to their typically developing counterparts. Parent report further distinguished autistic from non-autistic children. Machine-learning analysis combining all START-derived measures demonstrated 78% classification accuracy for the three groups. Qualitative analysis of the interviews with health workers and families of the participants demonstrated high acceptability and feasibility of the app. These results provide feasibility, acceptability and proof of principle for START, and demonstrate the potential of a scalable, mobile tool for assessing neurodevelopmental conditions in low-resource settings.

**Lay abstract:**

Autism is diagnosed by highly trained professionals– but most autistic people live in parts of the world that harbour few or no such autism specialists and little autism awareness. So many autistic people go undiagnosed, misdiagnosed, and misunderstood. We designed an app (START) to identify autism and related conditions in such places, in an attempt to address this global gap in access to specialists. START uses computerised games and activities for children and a questionnaire for parents to measure social, sensory, and motor skills. To check whether START can flag undiagnosed children likely to have neurodevelopmental conditions, we tested START with children whose diagnoses already were known: Non-specialist health workers with just a high-school education took START to family homes in poor neighbourhoods of Delhi, India to work with 131 two-to-seven-year-olds. Differences between typically and atypically developing children were highlighted in all three types of skills that START assesses: children with neurodevelopmental conditions preferred looking at geometric patterns rather than social scenes, were fascinated by predictable, repetitive sensory stimuli, and had more trouble with precise hand movements. Parents’ responses to surveys further distinguished autistic from non-autistic children. An artificial-intelligence technique combining all these measures demonstrated that START can fairly accurately flag atypically developing children. Health workers and families endorsed START as attractive to most children, understandable to health workers, and adaptable within sometimes chaotic home and family environments. This study provides a proof of principle for START in digital screening of autism and related conditions in community settings.

## Introduction

Autism is an early-onset neurodevelopmental condition with a global prevalence of ~1% (Zeidan et al., 2022). It is estimated that India is home to ~5 million families with a child with autism^1^ (Arora et al., 2018; Arun & Chavan, 2021; Chauhan et al., 2019; Patra & Kar, 2021; Rudra et al., 2017). Many of these children do not get diagnosed at an appropriate time, or at all, which in turn can reduce their chances to benefit from effective interventions (Divan et al., 2021). Low community awareness about autism leads to reduced help-seeking behaviour (Minhas et al., 2015), and is exacerbated by a number of other challenges to detection. First, there is a paucity of professionals, such as developmental practitioners, psychiatrists, neurologists and psychologists, to offer diagnostic services to a population of over 1.2 billion (Kumar, 2011). Second, current screening and diagnostic approaches typically involve time-intensive, expensive and proprietary tools, greatly limiting access (Durkin et al., 2015). While there have been notable efforts to develop locally validated instruments for screening and diagnosis, these too typically need to be administered by specialists (Gulati et al., 2019; Juneja et al., 2014; S. B. Mukherjee et al., 2015). Third, social stigma prevents parents’ seeking a psychiatric diagnosis for their child (Minhas et al., 2015).

Yet, there is emerging evidence from low- and middle-income country settings that non-specialist health-worker-delivered, parent-mediated intervention targeting social communication is acceptable and effective in improving outcomes for autistic children (Rahman et al., 2016). In light of such evidence, the detection gap becomes an urgent priority, highlighting the need for proactive screening for autism. The current study aimed at developing a tool usable by non-specialists to assess autism risk in low-resource settings, closing the detection gap.

Mobile technologies offer a significant advantage in this effort, given their wide penetration and scalability across geographies and socioeconomic strata. Similar efforts have shown promise in high-resource settings (Dawson & Sapiro, 2019; Egger et al., 2018). In the current study, we develop and provide the proof of principle for an online platform, consisting of a battery of tasks that index various aspects of the autistic phenotype, using a mobile device. In view of the diverse phenotypic domains associated with autism, the mobile platform (app) includes direct assessments of the child on multiple tasks that relate to social behaviour, sensory interest and motor function. The platform also includes an assessment of parent-reported autistic features through a questionnaire and an observational measure of parent–child interaction. While the broader aim of the project is to develop tools to bridge the detection gap for autism and related neurodevelopmental conditions, the current study constitutes the first step towards this goal by developing this tool and testing its efficacy and feasibility in a field study in children with and without neurodevelopmental disorders. To this end, we have implemented and benchmarked the assessment in the form of a scalable, mobile tool, administered in the community by non-specialists to assess autism-related features in 2- to-7-year-old children in home settings in India.

## Methods

**Figure 1.**
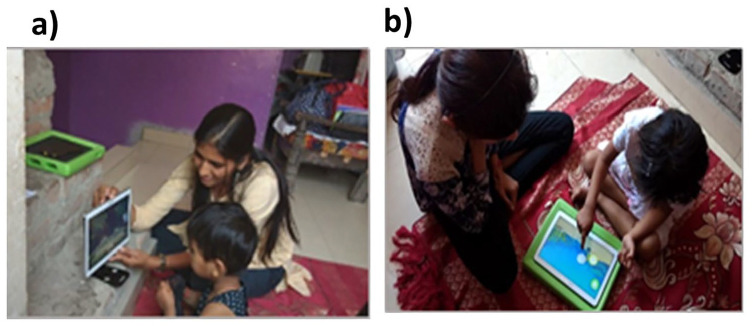
Administration of the START task battery in field settings. (a) Tablet positioned upright for the preferential looking task, and wheel task and (b) tablet positioned flat on a surface with a frame underneath for the button task, motor following task, bubble popping task and colouring task. Health workers and primary caregivers (in the case of children under 18 years of age), provided written informed consent for publication of the images in this figure.

**Figure 2.**
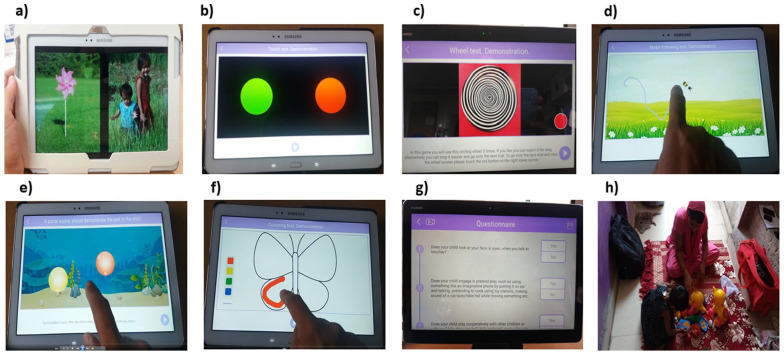
Sample screenshots from the (a) preferential looking task, (b) button task, (c) wheel task, (d) motor following task, (e) bubble popping task, (f) colouring task, (g) START questionnaire and (h) caregiver-child interaction observation. The primary caregiver provided written informed consent for publication of the image 2 h showing themselves and the child in this figure.

*Participants*: Three groups of children were recruited: (1) children with a diagnosis of Autism Spectrum Disorder (AS), *N* = 48; (2) children with a diagnosis of intellectual disability (ID), *N* = 43; and (3) typically developing (TD) children *N* = 40 (Table 1). The AS and ID groups were recruited through a tertiary clinic and diagnosed by a specialist clinician using Diagnostic and Statistical Manual of Mental Disorders (5th ed.; *DSM*-V) criteria, while the TD group was recruited from the community. All groups were matched for chronological age. The AS and ID groups were matched on cognitive age using a language-adapted version of the *Developmental Profile-3 (DP3)* (Alpern, 2007). The AS group was contrasted with the other two groups for the severity of autistic symptoms using a locally developed and standardised tool, the *INCLEN Diagnostic Tool for Autism Spectrum Disorder (INDT-ASD)* (Juneja et al., 2014). It is worth noting, however, that all children in the AS group also met criteria for ID. Written informed consent was obtained from a) the primary caregiver of each child participant, and b) each adult participant included for the qualitative data. Research participants/health workers/ primary caregivers (in the case of children under 18 years of age), provided written informed consent for publication of the images in Figures 1(a) and (b) and 2(h). All signed consent forms are stored in compliance with local confidentiality laws at the Child Development Group, Sangath, New Delhi, India.

**Table 1.** Participant characteristics.

	TD	AS	ID	F/χ2	*p*-value	Post hoc contrasts, *p*-value
Chronological Age *M* ± *SD*	(*N* = 40) 4.59 ± 1.34	(*N* = 48) 4.24 ± 1.22	(*N* = 43) 4.56 ± 1.67	*F(*2, 129) = 0.88	0.42	
Gender ratio (F:M)	19:21	12:36	9:34	7.99	0.02	
Cognitive age on DP3	(*N* = 36) 4.32 ± 1.49	(*N* = 37) 1.49 ± 0.53	(*N* = 36) 1.94 ± 0.80	*F*(2, 106) = 80.87	<0.001	TD > AS, <0.001 TD > ID, <0.001 ID ~ AS, 0.19
INDT-ASD	(*N* = 37) 0.16 ± 0.37	(*N* = 37) 17.16 ± 4.35	(*N* = 39) 5.15 ± 7.51	*F*(2, 110) = 109.97	<0.001	TD < AS, <0.001 TD < ID, <0.001 ID < AS, <0.001

AS: Autism Spectrum conditions; DP3: Developmental Profile 3; INDT–ASD: INCLEN Diagnostic Tool for Autism Spectrum Disorder; ID: intellectual Disability; TD: typically developing.

### Tools

The START (Screening Tools for Autism Risk using Technology) task battery was administered on all participants alongside two standardised tools for assessing autism symptom severity and developmental level. Details of these tools are given below:

The Developmental Profile 3 (DP3) (Alpern, 2007) is a parental interview scale designed to assess development and functioning across five areas: physical, adaptive, social-emotional, cognitive and communicative. We used the age-equivalent score from the cognitive subscale to estimate development that is not influenced by specific difficulties in social or communicative function.

The INCLEN Diagnostic Tool for Autism Spectrum Disorder (INDT-ASD) (Juneja et al., 2014) is specifically developed for diagnosing autism in 2- to 9-year-old children in India. It has a high validity against *DSM*-IV-TR diagnoses and Childhood Autism Rating Scale (Schopler et al., 1980) scores as well as with *DSM*-V (Vats et al., 2018).

The START task battery is an Android app presented on a mobile device that can be administered by non-specialists with minimal training. The app includes a battery of tasks that can be grouped within the following categories: social, motor, sensory, and parent/caregiver report and interaction (see Table 2). This choice of tasks was informed by the developmental differences commonly identified in autistic children. Social and sensory tasks are included to align with the two domains, social communication and restricted interests, by which autism is defined. These direct behavioural measures are complemented by activities to quantify parental observations and play-based interactions. More details of the phenotypic domains and tasks included in the battery are discussed in the sections below.

**Table 2.** Description of the tasks included in the START task battery.

Task	Relevant References	Task details	Stimuli	Procedure	Dependent variable
Domain: Social					
Preferential Looking Task (Figure 2(a))	Chakrabarti et al. (2017); Dubey et al. (2022); Hedger et al. (2018)	• Pairs of social and nonsocial videos were presented. • Size: each image covered half the screen in landscape mode leaving approximately 0.5 cm between the images. • No inter-trial interval or central fixation. • Counterbalanced presentation across the two sides of the screen. • Eight trials; total duration ~60 s.	• Social: Four videos of children looking towards the camera and smiling. • Nonsocial: Four videos of spinning washing machines or garden wind-fans.	• Tablet position: Upright on a stand (Figure 1(a)). • Setup: Child’s and tablet’s position were adjusted for a) camera alignment, b) light, c) distance, and d) stability. • Task: Child was instructed to look at the tablet screen and to keep their head still. • Child’s face was video-recorded using the front camera while s/he looked at the stimuli. • Rule to proceed: Once setup is completed, wait till the trial was completed	• Proportion of looking time to social stimuli
Button Task (Figure 2(b))	Dubey et al. (2022); Ruta et al. (2017)	• Two buttons were presented at random locations on the screen. • One button, when touched, showed the social video, and the other showed a non-social video. • The association between buttons and stimuli videos were counterbalanced between participants. • Eight trials; total duration ~120 s	• Social: a video of a child swimming and waving underwater. • Non-social: a video of a dynamic geometric pattern.	• Tablet position: Flat on the table (Figure 1(b)) with a soft frame underneath. • Demonstration: Child was shown that each button plays a video of either a child or a dynamic pattern. • Task: Child chose a button to play the linked video on each trial. • Rule to proceed: If the child successfully touched one button to start a video.	• The proportion of choices made to look at the social stimulus
Domain: Sensory Sensitivity					
Wheel Task (Figure 2(c))	Tavassoli et al. (2016)	• A video of a black and white wheel is presented on the screen. • A red button was presented in the right lower corner. This button could be pressed at any time to end the trial. • Five trials each lasting maximum of 15 s. • Total duration 75 s	• A 15-s video of a black and white wheel spinning to create a visual illusion.	• Tablet position: upright on a stand (Figure 1(a)). • Setup: Child was positioned in front of the screen. • Demonstration: Child was shown the task and instructed that they could press the red button any time they wanted to stop looking at the wheel. • Rule to proceed: If the child looked at the wheel carefully or tried to stop it by touching the red button. • Child’s face was video-recorded.	• Looking time at the video, calculated as a proportion of the duration for which the wheel videos were played, divided by the sum of the maximum duration for which the videos could be played if the ‘terminate’ button was not pressed. • Minimum and maximum distance between the child’s face and the tablet.
• Domain: Motor					
Motor Following Task (Figure 2(d))	Raw et al. (2012)	• A butterfly flying across the screen. • Random trajectories for the butterfly were generated with variable velocities in both the x and y axes. • Counterbalancing: the butterfly flew from left to right on two trials and right to left on the other two. • Four trials; total duration ~120 s.	• An image of a colourful butterfly over a background of a green field.	• Tablet position: Flat on the table with a soft protective cover underneath (Figure 1(b)). • Demonstration: Child was shown how to follow the butterfly across the screen by keeping the index finger of their dominant hand on top of the butterfly. • Task: Child followed the butterfly’s trajectory with their index finger. The child’s trajectory was displayed in real-time. • Rule to proceed: If the child was able to follow the butterfly making about ⅓ of its trajectory in the demonstration trial (maximum 3 attempts).	• Spatio-temporal error, jerk, and weighted frequency gain for the x and y axes.
Bubble Popping Task (Figure 2(e))	Anzulewicz et al. (2016)	• A series of bubbles were presented on the screen floating up and down in a straight line parallel to the y axis. • Six trials showing an increasing number (1 to 6) of bubbles on each trial. • Total duration: ~36 s.	• Images of bubbles on a colourful background of an underwater scene.	• Tablet position: Flat on the table with a soft protective cover underneath (Figure 1(b)). • Demonstration: Child was shown how to pop the bubbles and give a chance to do the same. • Task: Child was instructed to pop the bubbles as quickly as they could use one finger. • Rule to proceed: If the child popped two bubbles over a maximum of 3 demonstrations.	• Distance of touch from the centre of the bubble. • Force of touching the tablet.
Colouring Task (Figure 2(f))		• Simple outline figures and a colour palette were presented for colouring. • Two trials; maximum duration 240 s.	• Line figures of a flower and a butterfly. • Colour palette is presented at the left lower corner with red, yellow, blue, and green colours to choose from.	• Tablet position: Flat on the table with a soft protective cover underneath (Figure 1(b)). • Demonstration: Child was shown how to touch the palette to activate colour and move the finger to colour the figure. The child was given a chance to practise before starting the task. • Task: Child was asked to freely colour the figure. • Rule to proceed: If the child was able to make a stroke and go to the colour palette to pick a colour.	• Count of events crossing over the outlines of the target figure
Domain: parent/caregiver report and interaction					
START Questionnaire	Gulati et al. (2019); Vats et al. (2018)	• 14 binary choice (yes, no) items focussed on exploring the early signs of autism such as poor eye contact, limited social interactions etc. • Language used: Hindi	• Items adapted from INCLEN-INDT-ASD, M-CHAT, and ICD classification system.	• Tablet position: Held by a health worker. • Items were read aloud by the health worker.	• Each item is scored 1 or 0. Six items are coded as yes = 1, and no = 0. Eight items marked with (R) are reverse coded. The sum of scores is calculated across all items to index the severity of autistic symptoms.
Parent/Caregiver- Child Interaction (Figure 2(g))	Parent Child Interaction protocol from the Duke Centre for Autism and Brain Development (personal communication)	• A set of toys was presented to the caregiver and child to play with.	• Toys to facilitate pretend play, e.g. kitchen set, pretend food, cars, dolls, etc. Toys with lights and sounds to elicit sensory sensitivity. Toys to facilitate interaction and verbal outputs, e.g. toy phone, toy mobiles.	• Tablet position: The tablet was held by the health worker to record a video of the child and caregiver. • Video was recorded for 10 min.	• Synchronous responses from caregiver. • Initiation of interactions by child

INDT ASD: INCLEN Diagnostic Tool for Autism Spectrum Disorder; START: Screening Tools for Autism Risk using Technology; M-CHAT: Modified Checklist for Autism in Toddlers; ICD: International Classification of Disease.

All tasks on the app can be aborted using a unique gesture of sequential tapping on the corners of the tablet PC. All health workers were trained on the use of this gesture, with the instruction to abort a task in case a child did not engage with the tablet.

*Social phenotype*: Differences in social behaviour are a core diagnostic feature of autism. Lab-based experiments designed to measure this aspect of the autistic phenotype have often focussed on presenting social alongside nonsocial stimuli (Dubey et al., 2015; Pierce et al., 2011; Ruta et al., 2017). Such paradigms have revealed that autistic individuals have a reduced preference for social stimuli and make less effort to seek out social over nonsocial stimuli (Hedger et al., 2020). Accordingly, the START task battery includes two measures of social reward responsivity: 1) a passive viewing paradigm similar to the eye-tracking laboratory-based task of Pierce and colleagues (2011), and 2) a choice-based paradigm similar to that of Ruta and colleagues (2017). Reduced looking and responding towards social over nonsocial stimuli have been noted in autistic children in these prior reports. Accordingly, the key metrics of interest from these tasks were those that index the proportion of looking time or button presses towards social compared to nonsocial stimuli.

*Sensory phenotype*: Atypical sensory sensitivity is a commonly reported feature of autism (Ausderau et al., 2014; Ben-Sasson et al., 2007; Posar & Visconti, 2018). It is generally evaluated using parent-report questionnaires or tasks that involve touching/watching objects of special sensory interest (e.g. spinning wheels with illusory contours, pin cushions, musical dome). The START task battery includes an adapted version of one such lab-based task used by (Tavassoli et al., 2016) to measure visual sensory interest. In line with the key metric in the lab-based version of this task, the dependent variable of interest was the duration for which a child looked at the spinning wheel.

*Motor phenotype*: Atypical motor skills are commonly reported in autism (Anzulewicz et al., 2016; Ghaziuddin & Butler, 1998; Manjiviona & Prior, 1995). Poor spatial coordination and weak adaptation of velocity to reach targets have been suggested to be specific to autism (Forti et al., 2011). Developments in touch sensor technology can help measure spatial coordination and velocity with high precision and ease. The START task battery harnesses this technological development to measure three-dimensional finger movements, providing a fine-grained measure of spatio-temporal performance in fine-motor planning and execution. Three tasks were used to capture variability in motor performance: popping bubbles on a screen, following a butterfly across the screen with a finger, and colouring a pattern with clear outlines. The bubble popping task measured the force with which bubbles were popped as well as the distance of the touch from the centre of the bubble–in line with suggestions from previous research (Anzulewicz et al., 2016; Forti et al., 2011). Similarly, the motor following task measured the spatiotemporal errors in following a moving target, given the suggested autistic difficulties in motor coordination. Similarly, we measured the number of times that a child crossed over the boundaries of the figure in the colouring task, to provide a proxy for their motor control abilities.

Parent/Caregiver Report and Interactions: Behavioural observations may emerge from parent reports of day-to-day activities of the child, or expert observation of social interaction and play. Brief parent-report tools such as the INCLEN Diagnostic Tool for Autism Spectrum conditions (INDT-ASD) (Juneja et al., 2014), and All India Institute of Medical Sciences (AIIMS)-Modified-INDT-ASD Tool (Gulati et al., 2019) have demonstrated high sensitivity in early screening and diagnosis of autism in an Indian setting. Accordingly, the START app includes a brief questionnaire for primary care givers as well as a provision for video-recording a parent/caregiver-child play session. Dyadic interaction of the child with the caregiver constitutes one of the most ecologically valid metrics of social interaction, and is the primary target of certain types of developmental interventions for autism (Green et al., 2010). In line with previous reports, the key metrics of interest included the number of attempts by the child in initiating interactions, and the number of synchronous responses from the caregiver.

### Assessment procedure

Two high-school graduates with no prior relevant experience were recruited as non-specialist health workers. They underwent a 4-day training, with 2 days in classroom followed by 2 days of observation and supervised field-training in households. Two psychology postgraduate research assistants were recruited for the project to observe the data collection and ensure adherence to the research procedures. Each health worker was then paired with a research assistant to visit the participants’ houses to collect data, using a Samsung SM P600 tablet. Testing was generally conducted sitting on the floor or bed. Specialist assessment tools (DP-3 and INDT-ASD) were administered by the research assistants.

Research assistants completed a detailed observation schedule noting the environment and circumstances of each data collection, including family involvement and available resources. They interviewed non-specialist health workers both immediately after their training and at the end of data collection, with a focus on challenges faced during data collection and strategies adopted to overcome these. Research assistants interviewed parents of participating children (TD = 5, AS = 5, ID = 5) to explore their experiences with START, including caregivers of children who were able to complete the START assessment tasks and those who were unable to complete them. Separate consent for audio recording was taken prior to these interviews. Further details of the observation and interview schedules are available in the Supporting Information, sections 1.4 and 1.5.

All the procedures were performed in accordance with relevant guidelines and regulations and approved by the Research Ethics Committee of the University of Reading, UK, as well as the Institutional Review Board for Public Health Foundation of India, and the Indian Council of Medical Research.

## Community involvement statement

This project involved an autistic researcher who took part in regular discussions during the analysis of the pilot data collected using the START platform. In addition, the research team organised a dedicated dissemination and discussion event for the autism community stakeholders in India. It invited local clinicians, researchers, policymakers, and members of government organisations managing health care and disability support. The lead researchers gave a demonstration of the task battery to the audience and presented preliminary results in non-technical language.

## Analysis

The project used a mixed-methods design. Feasibility and acceptability were assessed via face-to-face interviews with non-specialist health workers and caregivers. The efficacy of the task battery in distinguishing children with neurodevelopmental conditions from other groups was quantified (using the general linear model [GLM]) for all dependent measures as functions of group. The evaluation of the task battery’s accuracy in classifying participants into the three groups applied machine learning methods including XGBoost, logistic regression, and support vector machines.

### Feasibility and acceptability

Interviews were conducted in Hindi with non-specialist health workers and caregivers to evaluate the feasibility and acceptability of the START task battery in home settings. Environmental conditions for data capture and the nature and frequency of disruptions during the assessment were recorded from the observation schedule used by the research assistant. All interviews were audio-recorded, transcribed, translated to English and cross-checked for accuracy of the translation. In-depth interviews were qualitatively analysed using thematic analysis (details in Supporting Information, Table S4).

### START app data analyses

Pre-set exclusion criteria were applied to the data to ensure quality, resulting in a different number of participants for each task. Detailed information for the analysis of each task and questionnaire measure within the app is provided below.

#### Preferential-looking task

Gaze location was identified using a convolutional neural net-based algorithm (Dubey et al., 2022; Krafka et al., 2016). Data were available from 118 of 131 participants (TD = 40, AS = 40, ID = 38). All participants met the inclusion criteria of eye detection for at least 50% of frames and gaze on the tablet for at least 50% of frames. Social preference was computed as a ratio between the number of frames during which a participant was gazing at the social stimulus and the total number of frames in which their gaze was identified to be on either of the two stimuli.

#### Button task

Data were available from 116 of 131 participants (TD = 40; AS = 37; ID = 39). Participants who completed fewer than 50% of trials were excluded, resulting in 104 participants (TD = 39; AS = 27; ID = 38) in the final analysis. For each participant, the proportion of social button choice as a fraction of the total number of completed trials was calculated.

#### Wheel task

Data were available from 125 of 131 participants (TD = 40, AS = 46, ID = 39). Participants who completed fewer than two trials or whose faces could be detected in only 25% or fewer of the video frames were excluded. This exclusion criterion yielded data from 117 of 125 participants (TD = 37, AS = 41, ID = 39) in the final analysis. Two variables were coded: a) Time spent looking at the wheel, and b) distance of the face from the screen. Time spent looking at the wheel was calculated for every completed trial, summed across trials, and divided by the maximum possible duration of the completed trials. The distance of the face from the screen was calculated using a deep neural network that detected the subject’s facial features in each frame (Bishain et al., 2021).

#### Motor following task

Data were available from 120 of 131 participants (TD = 40, AS = 43, ID = 37). Data sets were filtered for completeness by including only participants who finished two or more trials. This criterion yielded 115 participants (TD = 40, AS = 40, ID = 35) for the final analysis. Spatio-temporal difference between the target and the child’s motor trajectory was computed as root mean square error (RMSE) to measure accuracy in motor planning and execution. In addition, we analysed the ‘frequency gain’ metric for all participants using a Fast Fourier Transformation (FFT), to assess the closeness in the source and target motions along the frequency domain (for details, see Supporting Information, section 1.1).

#### Bubble popping task

Data were available from 120 of 131 participants (TD = 40, AS = 41, ID = 39). Data were included from all the participants who popped one or more bubbles. The force used while popping the bubbles was recorded using the getPressure() parameter recorded by the Android operating system on a Samsung tablet, and averaged across all bubbles popped. The distance between the touch point and the centre of the bubble was calculated to estimate visuomotor targeting accuracy in approaching dynamic stimuli.

#### Colouring task

Data were available from 113 of 131 participants (TD = 40, AS = 38, ID = 35). Participants were asked to colour the interior of a target figure. Data sets were included only if participants coloured at least 25% of the pixels on the screen. This criterion yielded 93 participants (TD = 37, AS = 29, ID = 27) in the analysis. The total number of crossings over the target figure’s outlines (movements in and out of the figure) was calculated. Any change in the touch point from inside the figure (pixels identified inside the outline) to the outside or *vice versa* was counted as one crossover.

#### Parent/caregiver–child interaction

Data were available from 100 of 131 participants (TD = 32; AS = 35, ID = 33). The video recording of the session was coded using the Dyadic Communication Measure for Autism, by three trained independent coders based in India (Green et al., 2010). Two measures were extracted from this data set, one indexing the child’s attempts at initiating interactions, and the other indexing synchronous responses from the caregiver. 13% of the videos were coded by all three coders and used to calculate intra-class correlation (ICC) using a 2-way mixed-effects model, based on a single measure, absolute agreement and confidence interval of 95%. A high degree of reliability was found between the coders for scores on parent/caregiver’s synchronous interaction as ICC was 0.876 (*p* < 0.0001, 95% CI [0.69, 0.96]). However, the coders had limited reliability for the scores on child’s initiation as ICC was 0.542 (*p* < 0.0001, 95% CI [−0.04, 0.85]). Where the videos were coded by more than one coder, we randomly chose codes from any one coder.

#### START questionnaire

Data were available from all 131 participants (TD = 40, AS = 48, ID = 43). The items were scored as binary responses. The summed score indicates the number of ‘red flag’ signs of autism.

For each task, the three groups were contrasted on the dependent variables defined above using analyses of variance (Table 3). The Kruskal–Wallis test was used where the assumption of normality was violated, and Welch and Brown-Forsyth robust tests were run where the assumption of homogeneity of variance was violated. Since the results from these alternative analyses were similar to those obtained with the general linear model, we report in Table 3 results from the standard analysis of variance. Results from the alternative statistical tests are presented in Supporting Information, Tables S2 and S3. In addition, we re-ran the analyses of variance reported in Table 3, including age as a covariate, which had no significant impact on the reported results. Since there were significant group differences in sex and cognitive age, we did not include these variables as covariates in this model.

**Table 3.** Group comparison using GLM for all measures from the START app.

Task	Dependent variable	TD mean (*SD*)	AS mean (*SD*)	ID mean (*SD*)	*F*	η2p	*p*	Post hoc comparison *p* value
Domain: social								
Preferential looking task	Social preference	*n* = 40 M = 0.59 (0.09)	*n* = 40 M = 0.52 (0.12)	*n* = 38 M = 0.53 (0.09)	*F*(2,115) = 5.996	0.09	0.003	TD > AS = 0.005 TD > ID = 0.025 AS ~ ID = 1.00
Button task	Social choice	*n* = 39 M = 0.47 (0.24)	*n* = 27 M = 0.52 (0.28)	*n* = 38 M = 0.52 (0.21)	*F*(2,101) = .638	0.01	0.53	NA
Domain: sensory								
Wheel task	Looking at the wheel	*n* = 37 M = 0.46 (0.37)	*n* = 41 M = 0.73 (0.33)	*n* = 39 M = 0.66 (0.36)	*F*(2,114) = 6.24	0.10	0.003	TD < AS = 0.003 TD < ID = 0.039 AS ~ ID = 1.000
Domain: motor								
Motor following task	RMSE	*n* = 40 M = 203.80 (97.95)	*n* = 40 M = 591.27 (283.63)	*n* = 35 M = 404.70 (216.74)	*F*(2,112) = 32.93	0.37	<0.001	TD < AS < 0.001 TD < ID < 0.0001 AS > ID < 0.001
	FFT X Axis	*n* = 40 M = 1.53 (.40)	*n* = 36 M = 2.01 (0.76)	*n* = 34 M = 2.02 (0.72)	*F*(2,107) = 7.21	0.12	0.001	TD < AS = 0.005 TD < ID = 0.005 AS ~ ID = 1.000
	FFT Y Axis	*n* = 40 M = 10.33 (9.12)	*n* = 36 M = 25.28 (15.42)	*n* = 34 M = 20.27 (11.51)	*F*(2,107) = 14.87	0.22	<0.001	TD < AS < 0.001 TD < ID = 0.002 AS ~ ID = 0.268
	Jerk	*n* = 40 M = .06 (.13)	*n* = 40 M = 0.05 (0.13)	*n* = 35 M = 0.05 (0.13)	*F*(2,112) = .053	0.001	0.948	
Bubble popping task	Force	*n* = 40 M = 0.07 (0.01)	*n* = 41 M = 0.09 (0.02)	*n* = 39 M = 0.08 (0.02)	*F*(2,117) = 8.49	0.13	<0.001	TD < AS ⩽ 0.0001 TD ~ ID = 0.140 AS ~ ID = 0.122
	Distance on x axis	*n* = 40 M = 45.20 (11.78)	*n* = 41 M = 88.97 (57.34)	*n* = 39 M = 66.81 (22.49)	*F*(2,117) = 14.54	0.20	<0.001	TD < AS ⩽ 0.001 TD < ID = 0.029 AS > ID = 0.023
	Distance on y axis	*n* = 40 M = 54.95 (13.59)	*n* = 41 M = 87.02 (43.17)	*n* = 39 M = 75.01 (25.03)	*F*(2,117) = 11.76	0.17	<0.001	TD < AS < 0.001 TD < ID = 0.011 AS ~ ID = 0.229
Colouring task	Crossing over	*n* = 37 M = 23.05 (16.98)	*n* = 29 M = 56.40 (29.43)	*n* = 27 M = 49.81 (28.31)	*F*(2,90) = 16.95	0.27	<0.001	TD < AS ⩽ 0.001 TD < ID < 0.001 AS ~ ID = 0.972
Domain: parent/caregiver report and interaction								
Parent/caregiver–child interaction	Caregiver: Synchronous response	*n* = 32 M = 0.33 (0.22)	*n* = 35 M = 0.15 (0.11)	*n* = 33 M = 0.20 (0.14)	*F*(2,97) = 11.46	0.19	<0.001	TD > AS ⩽ 0.0001 TD > ID = 0.004 ID ~ AS = 0.607
	Child: Initiation	*n* = 32 M = 0.48 (0.24)	*n* = 35 M = 0.23 (0.24)	*n* = 33 M = 0.40 (0.23)	*F*(2,97) = 9.94	0.17	<0.001	TD > AS ⩽ 0.0001 TD ~ ID = 0.516 AS < ID = 0.011
START questionnaire	Total score	*n* = 40 M = 1.03 (1.31)	*n* = 48 M = 5.06 (2.26)	*n* = 43 M = 3.09 (2.32)	*F*(2,128) = 44.06	0.41	<0.001	TD < AS < 0.001 TD < ID < 0.001 AS > ID < 0.001

AS: autism spectrum conditions; FFT: Fast Fourier Transformation; ID: intellectual disability; NA: not applicable; RMSE: root mean square error; *SD*: Standard deviation; START: Screening Tools for Autism Risk using Technology; TD: typically developing;

### Machine-learning analysis

This analysis applied a data-driven technique to combine the information from the multiple START metrics to optimise discrimination between the three groups (AS, ID, TD). Each dependent variable from the individual tasks constituted a feature vector. These features were then subjected to a set of machine-learning methods including XGBoost, logistic regression, and support vector machines. Each feature vector was first evaluated independently, and then in combination with other feature vectors for its accuracy in classifying individuals into the three groups (see Supporting Information, section 1.2, for details).

## Results

Results are presented below in three sections: a) feasibility and acceptability, b) group comparisons, and c) group classification accuracy using machine-learning analysis.

### Feasibility and acceptability

High completion rates (>70%) were obtained for all task measures collected (Supporting Information, Figure S2). The two main drivers behind missing data were a) children’s unwillingness to play a game, seen more often in atypical children compared to typically developing ones, and b) app malfunctions for specific tasks. While none of the children who did not complete a task had any documented visual, motor or auditory impairments, visual inspection of the data suggests that those who did not complete were more likely to be younger and of lower cognitive age than those who completed the tasks. Triangulation of data from the observation schedule and in-depth interviews highlighted the challenges in assessments such as limitations of space, variations in lighting, background noise and interruptions. Health workers identified the importance of the involvement of the family in meeting these challenges, and that of written standard operating protocols for guiding assessments. App-based assessment seems to have high acceptability for children, who actively played the ‘games’ on the tablet and enjoyed its child-friendly design elements. Parents also found START to be acceptable but questioned the credibility of an app-based assessment of child development (see Supporting Information, Table S4, for the list of themes).

### Group comparisons

We examined group differences in social, sensory, motor functions, parent/caregiver report and dyadic interaction. For each of these domains, the three groups were contrasted on the stated dependent variables (Table 3). In the social domain, an effect of group is seen on the preferential-looking task, as AS and ID children looked at the social stimuli less than the TD group did. However, no such group difference was seen in the button task. In the sensory domain, children with AS and ID looked at the spinning wheel longer than their TD counterparts did. In the motor domain, both AS and ID groups were distinguished from TD by force in the bubble-popping task and by visuomotor accuracy across all the motor tasks. Finally, an effect of group membership was found in measures of parent/caregiver report and interaction. Parents of autistic children endorsed higher numbers of items from the START questionnaire than parents of either ID or TD children. Inspection of Table 3 suggests a consistent pattern of difference between the two groups with neurodevelopmental conditions and the TD groups.

### Machine-learning analysis

**Figure 3.**
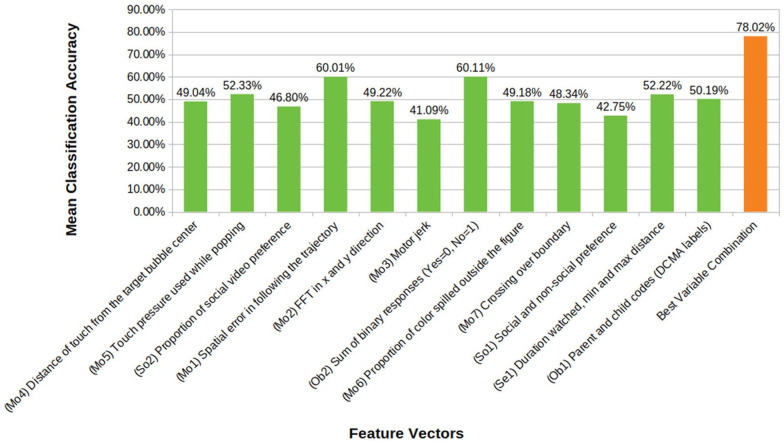
Mean classification accuracies of the feature vectors taken from the eight START tasks. The figure also represents the most accurate classification achieved by a combination of these features. (Prefixes on x axis in parentheses refer to corresponding feature IDs.) Some feature vectors are multidimensional amalgams of several different measures within a task. Chance level classification accuracy is 33.3%.

The classification accuracies, the sample proportions for each group and other details as determined in the machine-learning analysis are provided in Supporting Information, Table S1. Based on these results, the Motor Following task (RMSE in following the butterfly trajectory) was the most promising independent task with 60% overall classification accuracy into three groups (TD, ID, AS), superior to a random chance classification accuracy of approximately 33%. This discrimination accuracy is at par with that reported by the questionnaire measure (Figure 3).

While the classification accuracy for individual START metrics is relatively weak, combining the metrics yields a significant improvement, resulting in an overall classification accuracy of 78%, and an 86% accuracy in classifying typical development versus neurodevelopmental disorders in general (AS or ID) (Table 4). The combination of metrics yielding the most accurate classification consisted of the following: RMS error in the visuomotor following, boundary crossings in colouring, and force in bubble-popping; time watched and variation in distance from the display in the wheel task; both gaze and choice measures of social preference; and video-coded and questionnaire measures of autistic behaviour.

**Table 4.** Machine learning results. The overall classification accuracy for the best combination of feature vectors is listed. Refer to Figure 3 for corresponding Feature Vector IDs. So1: Button Task, So2: Preferential Looking task, Se1: Wheel task, Mo1: Motor Following Task, Mo5, Mo7: Bubble-popping task, Ob1: Parent Child Interaction, Ob2: Questionnaire responses.

Feature vector ID combination providing the best accuracy	Mean classification accuracy (AS)	Mean classification accuracy (ID)	Mean classification accuracy (TD)	Mean overall classification accuracy	Mean proportion % of subjects across different groups (AS: ID:TD)
Social: So1, So2 + Sensory: Se1 + Motor: Mo1, Mo5, Mo7 + Observation: Ob1, Ob2	61.61%	78.23%	86.40%	78.02%	23:30:47

AS: autism spectrum conditions; ID: intellectual disability; TD: typically developing.

## Discussion

We tested a battery of tasks, questionnaires, and observational measures administered by a non-specialist on a mobile platform (app) in three groups of children with and without neurodevelopmental conditions. This app was found to be both feasible for delivery by non-specialists in home settings and acceptable to all users including community health workers, parents, and children. We find strong evidence for group differences between children with and without neurodevelopmental conditions on most measures in the battery.

### Task measures

The task measures focussed on social, sensory and motor functioning. Specifically in the social domain, greater attention to social over non-social rewards was noted in non-autistic typically developing children. This pattern of results is consistent with reports on similar paradigms applied in laboratory settings, using standard infra-red eye trackers (Dubey et al., 2022; Hedger et al., 2020). In contrast to the preferential-looking task, the button task did not show a difference between the three groups. This absence of a group difference could be driven by differences in the administration of the task between the current and the original report on this paradigm (Ruta et al., 2017).

Strong group differences were noted in task measures of motor function. The non-autistic typically developing group performed more accurately than both the autistic and ID groups in the motor following task, as indexed by lower spatial errors (RMSE). Convergent findings indicating poorer visuomotor control in autistic children compared to the non-autistic typically developing group were demonstrated as greater numbers of boundary crossings in the colouring task, and lower accuracy in reaching a dynamic target in the bubble-popping task. In addition, the autistic group used significantly greater force than the non-autistic typically developing group in this task, replicating earlier reports (Anzulewicz et al., 2016). Greater force in hitting a target on the tablet as well as spatial targeting errors could be interpreted as a manifestation of poor motor control. Poor motor control can result from reduced use of sensory information to adjust motor behaviour and is consistent with theoretical models of sensorimotor and cognitive prediction error in autism (Van de Cruys et al., 2014)

In the domain of sensory interests, we used a tablet adaptation of a task previously associated with group differences between autistic and non-autistic children (Tavassoli et al., 2016). While the underlying mechanisms for enhanced interest in stimuli such as spinning wheels remain poorly understood, one feature shared by these stimuli is high predictability, which might be sought behaviourally as a mechanism to control sensory responsiveness or arousal. The current version of the task illustrates that autistic children show a similarly greater preference for the video of a spinning wheel, as indexed by a greater duration of looking at it compared to non-autistic children. In a phenotypic domain dominated by self- and parent-report instruments, this task shows promise as a scalable observational measure of visual sensory interests.

### Parent/caregiver-report and interaction measures

The parent/caregiver-report questionnaire was based closely on a tool specific for identification of autism in an Indian context (INDT-ASD). Unsurprisingly, scores on this questionnaire significantly differed between all three groups (AS, ID, TD) in the expected direction, replicating previous reports with the original tool (Gulati et al., 2019).

The caregiver-child videos revealed substantial atypicality in both key metrics of interaction. Autistic children initiated social interactions less than the TD group did, and also trended towards fewer initiations compared to the ID group. However, we advise caution in drawing strong inferences, since the inter-rater reliability for the child initiation behaviour was moderate. Fewer synchronous responses from the caregiver were evoked in interaction with both the groups of children with neurodevelopmental conditions (AS and ID), compared to those with TD children. This result is consistent with an earlier report of reduced synchronous parent–child interactions in autistic relative to TD children (Feldman et al., 2014).

The majority of the START measures showed the expected pattern of group differences between autistic children and their TD counterparts. These data demonstrate a) the feasibility of administering a multi-domain assessment of autism-relevant phenotypic dimensions at home by non-specialist health workers and b) the potential for scalability of this platform to other low-resource settings. However, we note the low specificity of these measures in discriminating between the AS and ID groups in the current sample. To investigate this apparent equivalence further, we re-examined each case’s clinical notes, which revealed that all of the autistic participants also met the criteria for ID. This observation reflects the ground realities in India, where most autism diagnoses in children within tertiary centres are at the severe end of the spectrum, and likely to be associated with developmental delay. In addition, a majority of the children in the ID group showed significantly elevated autistic symptoms. The phenotypic overlap in these groups likely contributed to the observed absence of group differences between AS and ID children for individual task metrics.

Notwithstanding this caveat, when combining all the measures to test their ability to discriminate the AS, ID and TD groups using machine learning, groups were classified with an overall accuracy of 78%, a considerable boost from the accuracy achieved by any of the measures alone. This level of classification accuracy is comparable to that achieved by machine-learning classifiers on structural brain imaging data, as well as the reliability of the autism versus other developmental conditions diagnoses by clinicians (Klin et al., 2000; Moon et al., 2019). This result highlights the advantages of a multi-measure platform that complements task performance with parent/caregiver report to achieve greater precision in assessing autism.

To our knowledge, this is the first demonstration of a multi-measure digital platform to assess autism-related symptoms by non-specialists in a low-resource setting. It adds to the growing number of international efforts towards digital assessments of autism (Dawson & Sapiro, 2019; D. Mukherjee et al., 2023). The largely non-verbal nature of the app’s directly assessed behavioural tasks makes it applicable in principle to other global settings without significant alteration. While we found that the START battery is sensitive to detecting deviations from typical development, individual task metrics did not clearly differentiate children with ID from those with AS. This observation is arguably driven by the nature of our sample of children with a neurodevelopmental disorder, where all autistic children met the criteria for ID, and several of the ID children had elevated autistic features. While this level of overlap is reflective of ground realities in our target population, and in any case makes an effective screening tool for neurodevelopmental disorders in general, future work can focus on validating this task battery further in neurodevelopmental disorder groups with minimal symptomatic overlap.

## Conclusion

The current study demonstrates the potential and proof of principle for a tablet-based app for assessing autistic children that can be administered by non-specialist health workers with minimal training. The app includes tasks, a questionnaire, and observational assessments of aspects of behaviour that index social, sensory, and motor function. Individual metrics from each task show a consistent pattern of differences between typically and atypically developing children. Combining the information from multiple measures within the app yields fairly accurate classification accuracy for the three groups of children (AS, ID, TD). Future work should test this app prospectively in a large population-based study to assess the predictive validity of these measures, independently and in combination, with atypical neurodevelopmental status.

## Supplemental Material

sj-docx-1-aut-10.1177_13623613231182801 – Supplemental material for Using mobile health technology to assess childhood autism in low-resource community settings in India: An innovation to address the detection gap Supplemental material, sj-docx-1-aut-10.1177_13623613231182801 for Using mobile health technology to assess childhood autism in low-resource community settings in India: An innovation to address the detection gap by Indu Dubey, Rahul Bishain, Jayashree Dasgupta, Supriya Bhavnani, Matthew K Belmonte, Teodora Gliga, Debarati Mukherjee, Georgia Lockwood Estrin, Mark H Johnson, Sharat Chandran, Vikram Patel, Sheffali Gulati, Gauri Divan and Bhismadev Chakrabarti in Autism

## Notes

1. We recognise that the autism community has a diversity of views in using person-first terminology. To reflect this diversity of views, we use ‘autistic children’ interchangeably with ‘children with autism’ throughout the article.
